# Pattern of pareses in 5q-spinal muscular atrophy

**DOI:** 10.1177/17562864241263420

**Published:** 2024-08-27

**Authors:** Zeljko Uzelac, Beate Schwäble, Johannes Dorst, Angela Rosenbohm, Kurt Wollinsky, Claudia D. Wurster, Janna S. Steinbreier, Albert C. Ludolph

**Affiliations:** Department of Neurology, Ulm University, Ulm, Germany; Department of Neurology, Ulm University, Ulm, Germany; Department of Neurology, Ulm University, Ulm, Germany; German Center for Neurodegenerative Diseases, Research Site Ulm, Ulm, Germany; Department of Neurology, Ulm University, Ulm, Germany; Department of Anesthesiology, RKU—University and Rehabilitation Clinics, Ulm University, Ulm, Germany; Department of Neurology, Ulm University, Ulm, Germany; German Center for Neurodegenerative Diseases, Research Site Ulm, Ulm, Germany; Institute of Human Genetics, Ulm University Medical Center, Ulm, Germany; Department of Neurology, Ulm University, Ulm, Germany; Department of Neurology, Ulm University, Oberer Eselsberg 45, 89091 Ulm, Germany; German Center for Neurodegenerative Diseases, Research Site Ulm, Ulm, Germany

**Keywords:** amyotrophic lateral sclerosis, motor neuron disease, paresis pattern, spinal muscular atrophy

## Abstract

**Background:**

This prospective study investigates the pattern of pareses in 5q-associated spinal muscular atrophy (SMA) to identify disease-specific characteristics and potential differences from amyotrophic lateral sclerosis (ALS) and spinobulbar muscular atrophy (SBMA). Detailed knowledge about pareses patterns in SMA facilitates differential diagnosis and supports therapeutic monitoring.

**Methods:**

Between January 2021, and June 2021, 66 SMA patients (59.1% male, aged 33.6 ± 15.2 years) were included in the study. Most patients had SMA type II (*n* = 28) or SMA type III (*n* = 28), seven patients had SMA type I, and three patients had SMA type IV. We analyzed the pattern of pareses using the UK Medical Research Council (MRC) scoring system.

**Results:**

In both, upper and lower limbs muscle weakness was less pronounced in distal (upper limbs: MRC median 3.0 (interquartile range 1.5–3.5); lower limbs: 1.5 (0.5–3.0)) compared to proximal muscle groups (upper limbs: 2.0 (1.5–2.6); *p* < 0.001; lower limbs: 0.5 (0.5–1.5); *p* < 0.001). Thenar muscles were stronger than other small hand muscles (3.0 (2.0–3.5) vs 3.0 (1.5–3.5); *p* = 0.004). Muscles had more strength in upper (2.3 (1.5–3.1)) compared to lower limbs (1.1 (0.5–2.3); *p* < 0.001) and in flexors compared to extensors.

**Conclusion:**

We identified a specific pattern of muscle paresis in SMA which is different from the pattern of paresis in ALS and SBMA. As a rule of thumb, the pattern of pareses is similar, but not identical to ALS in distal, but different in proximal muscle groups.

## Introduction

5q-associated spinal muscular atrophy (SMA) is an autosomal-recessive motor neuron disorder, clinically characterized by muscle weakness and atrophy caused by degeneration of alpha motor neurons due to a homozygous deletion or mutation in the survival motor neuron 1 (*SMN1*) gene. *SMN1* is located on chromosome 5q13 and is one of two genes encoding the SMN protein.^1,2^ The incidence of SMA in Europe is about 1/10,000 livebirths with a carrier frequency of 1/50.^3^ Based on the onset of clinical symptoms, the achievement of motor milestones, and life expectancy, SMA is divided into four subtypes (SMA type 0–4) according to the International SMA Consortium. Within the three main types (SMA type 1–3), SMA type 1 (Werdnig–Hoffmann disease) represents the infantile and thus most severe form, while SMA types 2 and 3 (Kugelberg–Welander disease) are defined as late-onset forms and are characterized by intermediate (SMA type 2) or mild (SMA type 3) types of progression. In SMA type 4, muscle weakness begins in the second or third decade of life.^4^

The severity of SMA phenotypes is determined primarily by the number of survival motor neuron 2 (*SMN2*) gene copies.^5^

Nusinersen, the first approved drug treatment option for SMA is an antisense oligonucleotide that increases SMN protein expression via modification of the splicing process of the pre-mRNA of *SMN2.*^6–8^ Risdiplam is a small molecule and oral medication acting in a similar way.^9,10^ Onasemnogene abeparvovec-xioi is a gene replacement therapy which comprises an adeno-associated viral vector with the human *SMN1* gene.^11–14^

The application of these disease-modifying treatment (DMT) leads to considerable improvements of muscle strength and motor function in SMA patients as demonstrated by clinical trials and real-world data.^14–20^ These effects are particularly evident in SMA patients treated early or presymptomatically,^20,21^ which has led to the implementation of SMA into newborn screening in many countries.^22^ The emergence of new SMA phenotypes under these therapeutic options is to be expected.^23^

The knowledge of a specific pattern of pareses is essential to better classify and monitor therapy-associated changes in motor function. It facilitates the differential diagnosis of SMA versus amyotrophic lateral sclerosis (ALS) and other motor neuron diseases in adults, for example, spinobulbar muscular atrophy (SBMA). Therefore, it was the goal of this study to determine the pattern of paresis in SMA and compare the results with ALS and SBMA.

## Methods

### Patients

Sixty-six patients were prospectively examined between January 2021 and June 2021 at the Department of Neurology of Ulm, University Hospital (Germany).

All patients had a genetically confirmed 5q-associated SMA with a homozygous deletion of exons 7, 8, or both, or with compound heterozygous mutations. The study was approved by the Ethics Committee of Ulm University (No. 19/12). Informed written consent was provided by all patients or their legal representative.

### Measurement of muscle weakness

Muscle strength was measured by the Medical Research Council (MRC) scale.^24,25^ The MRC is a standard scale for the quantification of muscle strength and has been used as a validated tool in a wide variety of clinical studies. Muscle strength is evaluated on a scale between 0/5 (no contraction), 1/5 (flicker or trace of contraction), 2/5 (active movement with gravity eliminated), 3/5 (active movement against gravity), 4/5 (active movement against moderate resistance), and 5/5 (full strength).

Muscle strength was evaluated in eight predefined muscle groups of upper and lower limbs, including elbow flexors and extensors, hand flexors and extensors, knee extensors and flexors, and foot elevators and flexors. Due to the high incidence of deformities and contractures preventing proper examination, muscle groups of shoulder and pelvic girdle were not tested.

### Statistics

For descriptive statistics, median and interquartile range (IQR) are given. For comparison of muscle groups, a two-step procedure was applied: First, differences between corresponding muscle groups on the right and left side of each limb were analyzed using the two-sided sign test. As this first step showed no significant differences between the right and left sides for all muscle groups, MRC measurements from both sides were pooled and analyzed collectively. Subsequently, the two-sided sign test was used to analyze differences between muscle groups. To investigate the differences between SMA types I, II, and III across various muscle groups, the Mann–Whitney *U* test was used. A result was considered as significant if the *p*-value was <0.05 (two-sided). Statistical analyses were performed using SPSS, version 26 (IBM Corp. Released 2019. IBM SPSS Statistics for Windows, Version 26.0. Armonk, NY: IBM Corp).

## Results

Sixty-six patients (59.1% male, aged 33.6 ± 15.2 years) were included. A total of 10.6% of patients were classified as SMA type 1, 42.4% type 2, 42.4% type 3, and 4.5% type 4. 6.4% had two copies of the *SMN2* gene, 63.8% had three copies, 27.7% had four copies, and 2.1% had only one copy. Fifty-four patients (81.8%) were treated with nusinersen and 12 patients (18.2%) with risdiplam. For a summary of patient characteristics see Table 1.

**Table 1.** Patient characteristics.

	Total	SMA 1	SMA 2	SMA 3	SMA 4
*N*	66	7	28	28	3
Sex (% male)	59.1	42.9	57.1	64.3	66.7
Age (years, mean ± SD)	33.6 (±15.2)	17.9 (±4.5)	26.4 (±11.3)	43.0 (±13.6)	49.0 (±9.6)
Copies of SMN2		2–3	2–4	2–5	4
Nusinersen (*N*, %)	54 (81.8%)	5 (7.6%)	20 (30.3%)	26 (39.4)	3 (4.6%)
Risdiplam (*N*, %)	12 (18.2%)	2 (3.0%)	8 (12.1%)	2 (3.0%)	0
HFMSE (median, range)			2 (0–19)	13 (2–66)	58 (58–62)
CHOP INTEND (median, range)		11 (3–25)	29 (1–39)		
6 min walking test in meters (median, range)				510 (353–690)	565 (556–648)

CHOP INTEND, Children’s Hospital of Philadelphia Infant Test of Neuromuscular Disorders; HFMSE, Hammersmith Functional Rating Scale Expanded; SMA, spinal muscular atrophy; SMN, survival motor neuron.

In some patients, certain muscle groups retained full strength (MRC grade 5). Elbow flexors, hand flexors, and hand extensors (in at least one arm) showed full strength in 12.1% of the patients, followed by foot flexors (5.3%), foot elevators (3.8%), knee extensors (2.3%), elbow extensors (1.5%), and knee flexors (0.8%).

**Figure 1.**
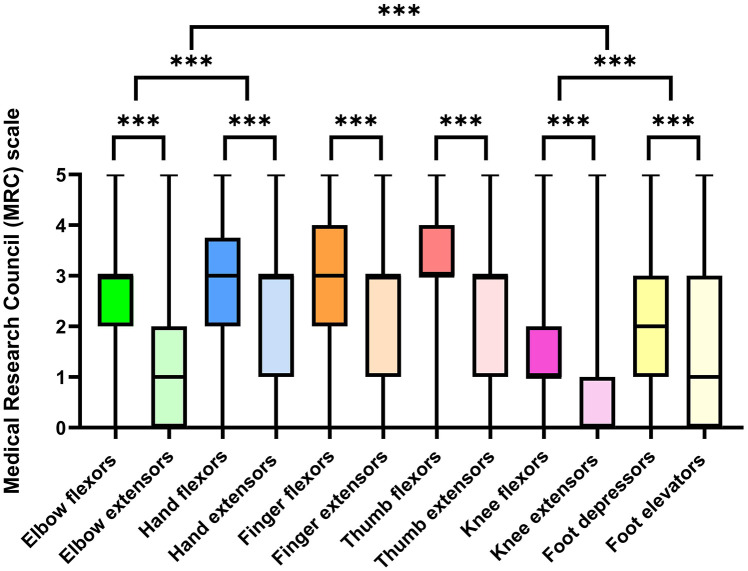
MRC scores for different muscle groups (median, minimum to maximum) and comparisons between muscle groups. ***p < 0.01. MRC, Medical Research Council.

Median and range of MRC scores obtained in different muscle groups are shown in Figure 1.

**Elbow** flexors (3.0 (2.0–3.0)) were stronger than elbow extensors (1.0 (0.0–2.0); *p* < 0.001), the same was true for **hand** flexors (3.0 (2.0–3.8)) if compared with hand extensors (3.0 (1.0–3.0); *p* = 0.004). Overall, hand muscles (3.0 (1.5–3.5)) were stronger than elbow muscles (2.0 (1.5–2.7); *p* < 0.001).

**Finger** flexors (3.0 (2.0–4.0)) were stronger than finger extensors (3.0 (1.0–3.0); *p* < 0.001) and **thumb** flexors (3.0 (3.0–4.0)) were stronger than thumb extensors (3.0 (1.0–3.0); *p* < 0.001). Overall, thumb muscles were stronger than muscles other fingers (3 (2.0–3.5) vs 3 (1.5–3.5); *p* = 0.004).

**Figure 2.**
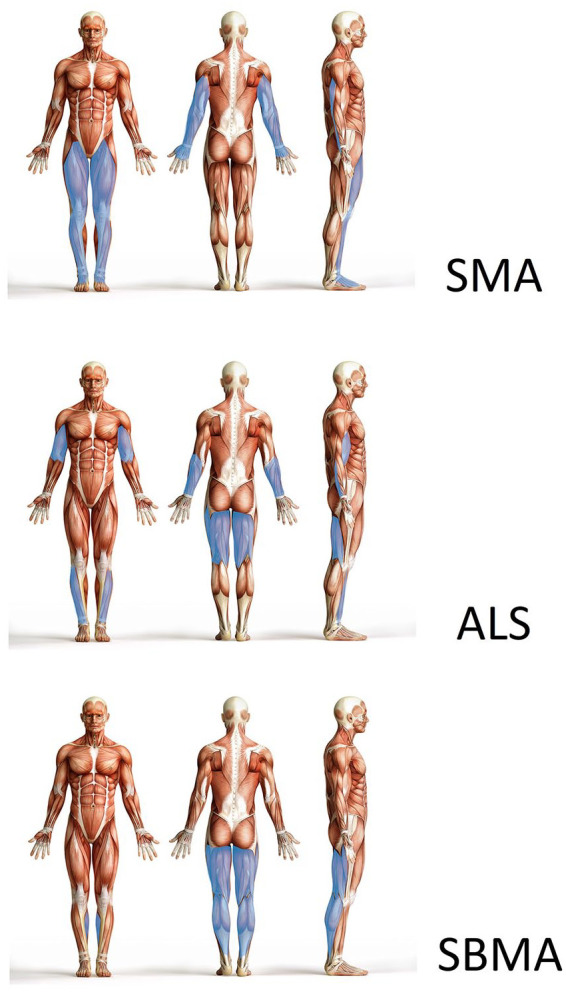
Pattern of pareses in SMA versus ALS versus SBMA. More affected muscle groups are marked with blue color. Compared to ALS, elbow and knee extensors are more severely affected in SMA, while elbow and knee flexors are less severely affected. In distal muscle groups, the pattern of paresis is similar. In SBMA, thigh and calf flexors are more severely affected than extensors. In arm muscle groups, all muscles are affected quite equally. ALS, amyotrophic lateral sclerosis; SMA, spinal muscular atrophy; SBMA, spinobulbar muscular atrophy.

**Figure 3.**
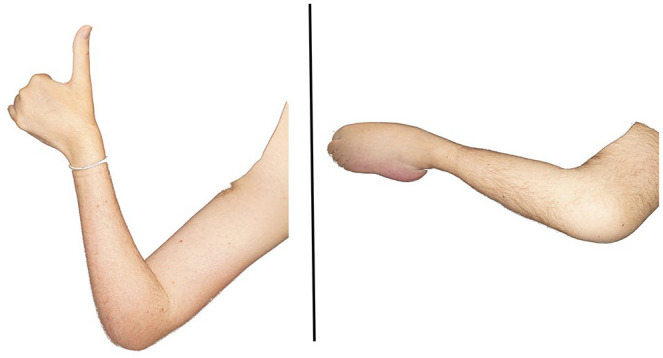
Pattern of muscle atrophy in SMA. Patient with SMA type 2, showing the typical pattern of pareses and atrophy of upper (left) and lower (right) extremities in SMA, predominantly affecting extensors, lower limb muscles, and proximal muscle groups. SMA, spinal muscular atrophy.

**Knee** flexors (1.0 (1.0–2.0)) were stronger than knee extensors (0.0 (0.0–1.0)); *p* < 0.001), and **foot** flexors (2.0 (2.0–3.0)) were stronger than foot elevators (1.0 (0.0–3.0); *p* = 0.001). Foot muscles (1.5 (0.5–3.0)) were stronger than knee muscles (0.5 (0.5–1.5); *p* < 0.001). On average, upper limbs (2.3 (1.5–3.1)) were stronger than lower limbs (1.1 (0.5–2.3); *p* < 0.001). In summary, compared to ALS, the pattern of pareses was similar in distal muscle groups, but opposite in proximal muscle groups (Figure 2). In SMA, lower extremities, proximal muscle groups, and extensors are predominantly affected (Figure 3).

The descriptive analysis of muscle group data for types I, II, and III of SMA showed that all SMA types shared a specific pattern of muscle weakness. Strength levels were lowest in SMA I, followed by SMA II, and were highest in SMA III (SMA I, II, and III; see Table 2). Significant differences in muscle strength were found in multiple areas. For the elbow, knee, hand, and foot muscles, strength was generally lower in SMA I compared to SMA II and III, and also lower in SMA II compared to SMA III, with statistical significance in most comparisons (*p*-values ranging from <0.001 to <0.02).

**Table 2.** Descriptive analysis of muscle groups data by types 1–4.

Muscle group	SMA type 1		SMA type 2		SMA type 3		SMA type 4	
*N*	7		28		28		3	
	Mean	SD	Mean	SD	Mean	SD	Mean	SD
Elbow extensors	0.5	0.1	0.7	0.2	2.1	1.4	3.0	0.0
Elbow flexors	1.4	1.2	2.4	1.0	3.3	1.0	5.0	0.0
Hand extensors	1.2	1.1	1.5	1.2	3.6	1.0	4.3	0.8
Hand flexors	1.3	1.1	2.3	1.2	3.4	1.0	4.5	0.5
Finger extensors	1.2	1.1	1.6	1.0	3.3	0.8	3.8	0.8
Finger flexors	1.7	1.1	2.4	1.1	3.6	1.0	4.2	0.8
Thumb extensors	1.0	0.5	2.1	1.1	3.2	0.8	3.5	0.5
Thumb flexors	1.7	0.9	2.6	0.9	3.8	0.8	4.5	0.8
Knee extensors	0.1	0	0.3	0.2	1.1	1.0	2.7	0.5
Knee flexors	1.0	0.6	1.1	0.6	2.0	1.1	3.7	0.5
Foot elevators	0.2	0.1	1.2	1.1	2.6	1.6	4.0	0.6
Foot depressors	0.9	0.4	1.5	1.0	2.6	1.2	3.8	0.8

SMA, spinal muscular atrophy.

However, no significant differences were found in muscle strength between SMA types I and II for the elbow extensors, hand extensors, knee flexors, and knee extensors (*p* > 0.05).

## Discussion

Our findings support the assumption of a specific pattern of pareses in SMA. This pattern is characterized by a pronounced muscle weakness of lower limbs and preferentially proximal muscle groups.

The pattern is distinctly different from ALS, the most frequent motor neuron disease in adults. In ALS, pronounced weakness in thumb muscles, hand/finger extensors, elbow flexors, knee flexors, and foot extensors is a specific diagnostic feature of the disease.^26^ Also typical is the paresis pattern of the intrinsic hand muscles which involve pronounced weakness and atrophy of the abductor pollicis brevis (APB) and first dorsal interosseous (FDI) muscles with relative sparing of the abductor digiti minimi (ADM) (“split-hand” syndrome).^27^ Cortical influences, in particular a pathogenetic role of monosynaptic corticomotoneuronal (CM) input, have been suggested to explain this pattern.^27–30^ Accordingly, it is reasonable to assume that in ALS muscle groups receiving the strongest direct CM innervation by phylogenetically young monosynaptic inputs are most severely affected.^26,31–35^

In SBMA, proximal legs are more affected than arms, and the tongue shows a pattern distinguishable from ALS with a higher fat fraction in muscle magnetic resonance imaging (MRI).^36^ From a clinical point of view, severe tongue atrophy in SBMA goes along with a striking lack of dysarthria. Also, the lower limbs in SBMA show a higher amount of fatty infiltration compared to ALS.^36^ In the thigh, a relative sparing of the medial muscle group (adductor magnus, sartorius, soleus) with early involvement of the posterior thigh muscles and quadriceps are observed. In the lower leg, the posterior deep and superficial calf compartment (gastrocnemius, soleus, and tibialis posterior) is predominantly affected with a relative sparing of tibialis anterior. These MRI results correlate well with the functional rating scales (lower limb part of ALS Functional Rating Scale – Revised and SBMA Functional Rating Scale) in Klickovic et al.^36^ Regarding the paresis pattern in the arms, involvement is more diverse: According to literature, finger extensors and elbow flexors are most commonly affected,^37^ but distal and proximal arm muscles (extensors and flexors) are almost equally affected. In contrast to ALS, SBMA involves not only motor neuron degeneration but also considerable muscle involvement which may partly explain the distinct muscle vulnerability and paresis pattern.^38^

In contrast to ALS, very few studies examined the specific pattern of pareses in SMA. In line with our study, Günther et al.^39^ showed that adult SMA patients suffer from a “reversed split-hand” phenomenon, which discriminates SMA from ALS and controls with a high sensitivity and specificity. In their electrophysiological study using Motor Unit Number Index (MUNIX),^39^ the most affected muscle in SMA was the FDI, followed by the ADM, whereas the APB was relatively well preserved. There are reports of patients with other pure lower motor neuron diseases, who have also been described as having a “split-hand.”^40,41^ In patients with spinal and bulbar muscular atrophy (SBMA, Kennedy’s disease), a “split-hand” was described in 57% of patients.^42^ We assume that the “reversed split-hand” phenomenon might represent a specific pattern of paresis in SMA and can therefore help to clinically differentiate SMA from ALS and other motor neuron diseases in adults.

The pattern of paresis in proximal and distal muscle groups of upper and lower extremities can also be utilized to distinguish adult-onset SMA from ALS and SBMA. As a rule of thumb, the pattern of paresis is similar in distal muscle groups (apart from the hand patterns), but differs in proximal muscle groups. In both SMA and ALS, the typical pattern of paresis in distal muscle groups involves a pronounced affection of finger/hand extensors and foot elevators. On the other hand, elbow and knee extensors are more severely affected in SMA, whereas elbow and knee flexors are more severely affected in ALS. In SBMA, the posterior leg flexors in the thigh and lower leg are more severely affected, while in the less affected arms, a specific pattern for flexors or extensors could not be determined in proximal and distal muscle groups.

As this was an observational study, we were unable to analyze the effect of specific SMA treatments such as nusinersen and risdiplam, on paresis patterns. However, our findings mith impact future longitudinal studies which assess the longitudinal impact of SMA therapies. Based on our results, we suggest a nuanced approach for monitoring treatment effects, focusing on muscles with moderate function (power 3–4) rather than those with severe weakness (power 1–2). This might offer a more sensitive measure of therapeutic efficacy, as these muscles show more significant and measurable responses. Similarly, CMAP assessments in muscles with moderate pareses will likely provide more meaningful data, reflecting subtle functional improvements and serving as more robust indicators of treatment success.

The emergence of new SMA phenotypes under DMT is to be expected; the question remains whether the pattern of paresis described here might change in the future, or whether treatment will only exert quantitative effects.

## Declarations

**Ethics approval and consent to participate:** The study was executed in accordance with the Declaration of Helsinki and was approved by the Ethics Committee of the University of Ulm (Nr. 19/12). Informed written consent was provided by all patients.

**Consent for publication:** Not applicable.

**Competing interests:** C.D.W. has received honoraria from Biogen as an advisory board member and for lectures and as an advisory member and consultant from Hoffmann-La Roche. She also received travel expenses from Biogen. A.C.L. received financial research support from AB Science, Biogen Idec, Cytokinetics, GSK, Orion Pharam, Novartis, TauRx Therapeutics Ltd, and TEVA Pharmaceuticals. He also has received honoraria as a consultant from Mitsubishi, Orion Pharma, Novartis, Teva, and as an advisory board member of Biogen, Treeway, and Hoffmann-La Roche. A.C.L. is Associate Editor at Therapeutic Advances in Neurological Disorders; therefore, the peer review process was managed by alternative members of the Board and the submitting Editor was not involved in the decision-making process. J.D. has received honoraria from Biogen as a consultant. A.R. has received honoraria as a consultant from Amicus Therapeutics, Sanofi, Hormosan, and Fulcrum. Z.U. has received honoraria and grants from Biogen as a consultant. K.W., B.S., and J.S.S. report no disclosures.

**Data access, responsibility, and analysis:** Z.U. has full access to all the data in the study and takes responsibility for the integrity of the data and the accuracy of the data analysis. Z.U. conducted and is responsible for the data analysis.

**Data sharing:** Individual participant data that underlie the results reported in this article, after de-identification (text, tables, and figures), as well as the study protocol will be available. Data will be available beginning 3 months and ending 5 years following article publication. Data will be shared with researchers who provide a methodologically sound proposal. Data will be shared for analyses to achieve the aims in the approved proposal. Proposals should be directed to zeljko.uzelac@rku.de; to gain access, data requestors will need to sign a data access agreement. Data are available for 5 years at https://www.uniklinik-ulm.de/neurologie.html.

**Availability of data and materials:** Not applicable.
